# Facial Anti-Spoofing Using “Clue Maps”

**DOI:** 10.3390/s24237635

**Published:** 2024-11-29

**Authors:** Liang Yu Gong, Xue Jun Li, Peter Han Joo Chong

**Affiliations:** Department of Electrical and Electronic Engineering, Auckland University of Technology, Auckland 1010, New Zealand; xuejun.li@aut.ac.nz (X.J.L.); peter.chong@aut.ac.nz (P.H.J.C.)

**Keywords:** anti-spoofing detection, Swin Transformer, ResNet, auto-encoder

## Abstract

Spoofing attacks (or Presentation Attacks) are easily accessible to facial recognition systems, making the online financial system vulnerable. Thus, it is urgent to develop an anti-spoofing solution with superior generalization ability due to the high demand for spoofing attack detection. Although multi-modality methods such as combining depth images with RGB images and feature fusion methods could currently perform well with certain datasets, the cost of obtaining the depth information and physiological signals, especially that of the biological signal is relatively high. This paper proposes a representation learning method of an Auto-Encoder structure based on Swin Transformer and ResNet, then applies cross-entropy loss, semi-hard triplet loss, and Smooth L1 pixel-wise loss to supervise the model training. The architecture contains three parts, namely an Encoder, a Decoder, and an auxiliary classifier. The Encoder part could effectively extract the features with patches’ correlations and the Decoder aims to generate universal “Clue Maps” for further contrastive learning. Finally, the auxiliary classifier is adopted to assist the model in making the decision, which regards this result as one preliminary result. In addition, extensive experiments evaluated Attack Presentation Classification Error Rate (APCER), Bonafide Presentation Classification Error Rate (BPCER) and Average Classification Error Rate (ACER) performances on the popular spoofing databases (CelebA, OULU, and CASIA-MFSD) to compare with several existing anti-spoofing models, and our approach could outperform existing models which reach 1.2% and 1.6% ACER on intra-dataset experiment. In addition, the inter-dataset on CASIA-MFSD (training set) and Replay-attack (Testing set) reaches a new state-of-the-art performance with 23.8% Half Total Error Rate (HTER).

## 1. Introduction

Since facial recognition is widely applied in many fields of daily life (e.g., accessing personal accounts via facial identification), face spoofing is becoming a big threat to users and making some online systems vulnerable. The presentation attacks (PAs), such as paper print attacks, replay attacks, and 3D facial masks are widely used and easily controlled by hackers. Thus, developing a reliable facial anti-spoofing (FAS) method [1,2,3,4] is important to avoid security risks and financial losses.

Among the proposed FAS methods, handcrafted feature-based [5] and deep learning-based methods [6] are common, and these methods are popular in processing single-modal data (RGB images). However, those models that only utilize hand-crafted features are not reliable, with low representation capacity due to different novel types, and some physiological signals [7] and handcrafted clues cannot be effectively learned by only spoofing images with a single model. Therefore, one major method is to combine multiple modalities of data such as depth and infrared (IR) images or design an ensembled system to distinguish spoofing attacks, which is the mainstream to reach the state of the art. On the other hand, another approach is to create a more advanced model architecture and combine different learning methods to extract as rich and effective spoofing features as possible. As some datasets cannot provide corresponding depth or physiological information, it is undeniable that this type of method is rather promising and challenging in future work.

Enlightened by the approach to combining different learning methods, this paper proposes a novel Encoder–Decoder face anti-spoofing structure which utilizes Transformer as the Encoder and ResNet as the Decoder. The total loss is determined by three loss functions instead of using cross-entropy loss only. In the Encoder part, we only utilize the Swin Transformer as an extractor with an auxiliary classifier to achieve preliminary classification work. In addition, we assume the Transformer-based feature extractor could extract more useful features with correlations of patches, the combination of softmax and cross-entropy loss is used directly after applying L2 Normalization on extracted features. In the Decoder part, we regard this part as a “Clue Map” generator, the proposed method applies a semi-hard triplet loss [8] to minimize intra-class sample distances and maximize the inter-class sample distances first. This is useful to find out the minor differences between spoof images and live images. The main purpose of the Decoder is to revert the extracted features in the latent space to the “Clue Map” for live and spoof images and we apply pixel-wise loss to supervise them. Because live images should not have any “spoof clues”, thus, the generated Clue Maps for live images should tend to be all-zero maps. In contrast, the generated Clue Maps for spoofing images should tend to be all-one maps. Even though the proposed work is based on the existing works, this method is a new attempt that combines Swin Transformer and ResNet to design a new model architecture. Based on the experimental results, it is proven to be robust and could enhance performance compared with previous methods. In addition, few papers apply the Swin Transformer as a feature extractor in the FAS field, and our preliminary work compares Swin Transformer with other Convolutional Networks and proves that Swin Transformer also has excellent spoofing feature extraction capabilities on Face Anti-spoofing. In addition, the generated “Clue Maps” by our proposed methods could directly be applied in other unsupervised learning such as consistency calculation or contrastive learning methods. In conclusion, the main contribution of this work is threefold:

(1) Design an Auto-Encoder structure based on Swin Transformer and ResNet. The Swin Transformer aimed to extract features with patch correlation and ResNet aimed to generate the corresponding “Clue Maps”.
(2) Combine cross-entropy loss, and semi-hard triplet loss with Smooth L1 pixel-wise loss to supervise the spoof detector, which has proven to increase the model’s generalization ability.
(3) Compared with some feature fusion methods, it could reach a new state-of-the-art performance, and the generated Clue Maps could be utilized in future contrastive learning methods.

## 2. Related Work

In this section, we revisit several representative anti-spoofing methods, which are divided into two main categories: convolutional neural network (CNN) anti-spoofing and hybrid learning methods. The CNN anti-spoofing methods usually include a direct supervised learning framework, pixel-wise supervision, and generative models. However, hybrid learning methods always combine hand-crafted features with CNN-extracted features. The main research directions and trends are more inclined to develop different feature fusion methods. Finally, we make a summary of the proposed backbone: the Swin Transformer as well.

### 2.1. CNN Anti-Spoofing Detection

Advanced end-to-end CNN models could effectively map input images to spoofing detection, and most previous work used binary cross-entropy loss for direct supervised learning. These methods are efficient to use because cross-entropy loss could supervise Face Anti-Spoofing work to converge faster. But it easily causes the model to overfit and the reals and Presentation Attacks always hold asymmetric distribution [9], which makes models struggle to learn a latent space with smaller intra-class distance and larger inter-class distance between samples. For example, Xu et al. [10] designed a fine-grained classification network that contains different spoofing attack methods. This multi-class supervision work could represent some detailed properties (paper attack and replay). On the other hand, applying different losses, such as adopting focal loss or angular margin softmax loss, is also useful in solving hard sample challenges and is widely used in some anti-spoofing research. The second method is pixel-wise supervision with an auxiliary classifier, some PAs do not have facial depth information, so generating pixel-wise pseudo labels [11] could be regarded as discriminative supervision signals. Then, the pseudo-labels enforce the models to predict the depth maps for live images while the zero-maps for spoof ones. Overall, pixel-wise supervision is beneficial for explaining feature learning and reaching a higher evaluation performance. However, the main limitation is the high demand for training data resolution.

### 2.2. Hybrid Learning Methods

**Figure 1:**
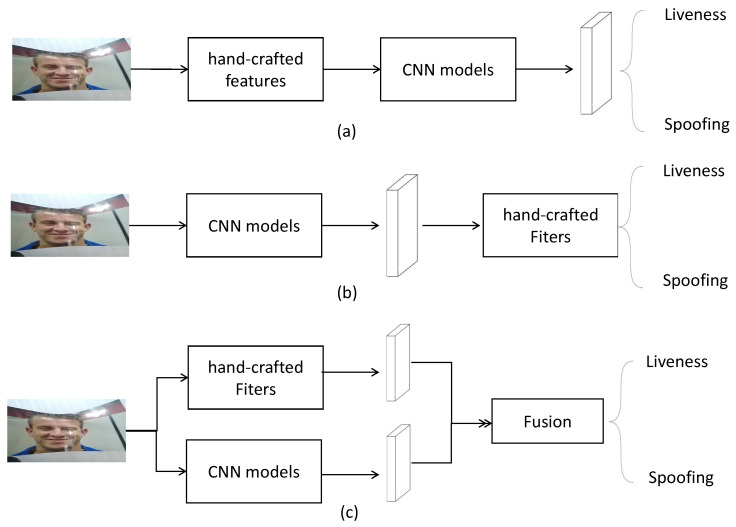
Three common hybrid learning methods for FAS. (**a**) represents CNN features from hand-crafted features. (**b**) represents CNN features filtered by hand-crafted methods. (**c**) represents feature fusion of hand-crafted features and deep learning features.

The hybrid learning method is described as a technique combining hand-crafted features with deep learning features, because hand-crafted features such as HOG [12] descriptors have proven to be strongly discriminative in some commercial RGB cameras in real life. There are three main hybrid learning approaches (see Figure 1) in the previous studies. Firstly, they extract hand-crafted features from the original input faces, and then send them to a deep-learning network. For example, Khamari [13] extracted LBP and Weber descriptors and then encoded them with CNN features which aims to obtain local intensity and edge information with semantic features. However, using hand-crafted methods at the initial stage will lose pixel-wise information, which causes low performances of these models. Secondly, some approaches choose hand-crafted methods to filter out irrelevant deep features. Li et al. [14] used Principle Components Analysis (PCA) to reduce unrelated redundancy of deep features extracted from the VGG face model. Although traditional hand-crafted methods increase the discriminative ability, the semantic representation of the overall model degrades to some extent. The last method is to fuse hand-crafted features with deep features together and send to binary classification which is the commonly used method in recent studies.

### 2.3. Swin Transformer

Convolutional Neural Networks such as ResNet [15] and Xception [16] are widely applied in image classification work; however, Vision Transformers [17], inspired by the self-attention mechanism [18], reached a new domain on distinguishing images in the computer vision field, which split images into several patches and combined their correlations. Swin Transformer [19] is a hierarchical architecture with shifted windows and has become increasingly powerful among different Vision Transformer (ViT) models. The main contribution of the Swin Transformer is to design a shifted window and apply a cyclic shift to obtain the interaction information between separated patches, which is unlike computing global self-attention by other ViTs. This could largely solve the shortcomings of some traditional vision transformers that do not have overlapping image patches. On the other hand, patch merging performs an important role in building hierarchical feature maps by adjusting each stage feature’s resolution and channels. Swin Transformer reduces computation complexity and proves that it could be a general backbone for dense recognition tasks. In ImageNet classification, this approach currently has state-of-the-art accuracy with appropriate FLOPs and parameters.

To prove that the Swin Transformer could effectively extract the spoofing features, we only utilize the Swin Transformer as a feature extractor and directly make a binary classification on RGB images of CASIA-MFSD. Then, we chose an Equal Error Rate (EER), which is the point on the ROC curve that corresponds to having an equal probability of miss-classifying a positive or negative sample, then we compared with some CNN-based methods and handcrafted methods, the results are shown in Table 1.

By the preliminary experiments, we can conclude that the Swin Transformer could work more effectively than some traditional backbones and handcrafted methods. However, depth maps could examine whether the inputs have face-like depth information, which largely enhances models’ performances in the current stage.

## 3. Proposed Methods

In this section, we will explain our work from three aspects: data pre-processing, network design, and loss function design. Among them, the data pre-processing part only localizes, aligns, and crops human faces, and finally, the random geometric data augmentation methods are applied to the images obtained. The purpose of network design is to extract more variety of features and restore them to the corresponding Clue Maps. The last stage is to more effectively regulate the difference between the predicted value and the Ground Truth labels.

### 3.1. Data Pre-Processing

**Figure 2:**
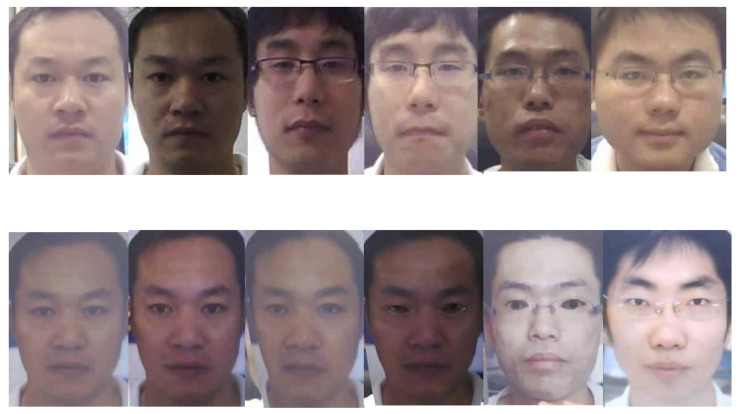
The cropped samples of CASIA-MFSD detected by facial area detector, the first row pictures are all liveness faces shot by different cameras, the second-row images include paper attack, paper mask and replay attack.

This method is designed based on extracting spatial information features of images; thus, we need to extract video frames and locate face position information for specific datasets such as CASIA-MFSD [4]. Since there is no obvious change in the expression and postures of the human faces in most video frames, we choose to calculate the total number of video frames in each video and obtain three video frames from each video to prevent the duplication of facial content information. At the same time, we reprocess the provided bounding box information of the OULU dataset [22] and use the facial detector to obtain facial areas of original data in CASIA-MFSD. Then, we expand the width and height of the face area by 20% to ensure that the edge information of the face is not lost. After we applied facial alignment and cropped facial areas, the samples (see Figure 2) were sent to random data augmentations to enrich input varieties, which is the first stage to avoid overfitting in our design. Specifically, we set a confidence threshold of 0.5 and a Non-Maximum Suppression (NMS) as 0.5 for facial detector “scrfd”. There are several parameters we set for the data augmentations. Firstly, we selected the random data augmentation methods, which combine base transformation, horizontal flipping, vertical flipping, random rotation, and random resize crop. Then, there is a probability of 0.8 that vertical and horizontal flipping will flip the original input images. Additionally, we set the rotation angle from 60 degrees to 90 degrees for random rotation augmentation to increase the input varieties. Finally, the Random resize Crop scale is from 0.77 to 1 and the ratio is from 0.9 to 1.1.

### 3.2. Network Architecture

**Figure 3:**
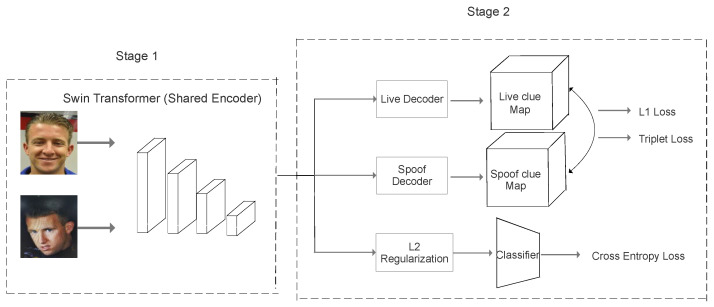
The proposed network architecture. The Transformer-based Encoders have shared parameters, but Decoders are trained separately and designed based on the ResNet structure with 27 convolutional layers without linear layers.

The proposed network architecture which consists of a shared parameter Transformer-based Encoder (Stage 1) and two ResNet-based Decoders (Stage 2) is illustrated in Figure 3. The main purpose of the Encoder (Stage 1) is to extract features and then save them into latent space; Stage 2 can be divided into three main branches: two Clue Map generators (Decoders) and one binary auxiliary classifier. The main purpose of designing the entire model framework as an Auto-Encoder with an auxiliary classifier is avoiding fully supervised learning for the training model. Auto-Encoder is an unsupervised learning model in nature which could largely reduce the dependence of data on annotations. In addition, the input data for the entire model has already been compressed into low-dimensional representations and stored in the latent space before passing through the Decoder part. This could allow the most important feature information to be extracted and minimize the impact of noise on the recognition model. Another benefit of Auto-Encoder is that its architecture is flexible and is not limited to the number of layers of the network model. Additionally, the latent representations can be used for classification work or clustering.

In Stage 1, the input images are firstly split into several non-overlapping patches, and regarded the patches as “tokens”, which is the same as the operation of other Vision Transformers. Then, the corresponding patch size is set as 7×7, and patch embeddings are processed by 12 Swin Transformer blocks with self-attention computation to obtain features *f* with the size of [7,7,768]. Specifically, the query (*Q*), key (*K*) and value (*V*) are input vectors after applying linear projection on the embeddings, the query and key are with the same vector dimension of *d*. By computing the dot product of the query and key and adding bias, we utilize the Softmax function to squeeze the output range from 0 to 1. The corresponding self-attention computation is written as in Equation (1). All features *f* are saved in the latent space, and Stage 2 is to further process these saved features. In addition, we set the dropout rate as 0.8 in Multiple Layer Perceptron (MLP) to avoid over-fitting in this stage. After obtaining the original output features extracted by the Encoder, we reshape the tensor size to two-dimensional, which is [49,768].

(1) $$Attention\left(Q,K,V\right)=Softmax\left(QK^{T}/\sqrt{d}+B\right)V$$

where query (*Q*), key (*K*), and value (*V*) are obtained from linear projection; $1/\sqrt{d}$ is the scale factor of query and key; and *B* is the bias of query, key, and value.

**Figure 4:**
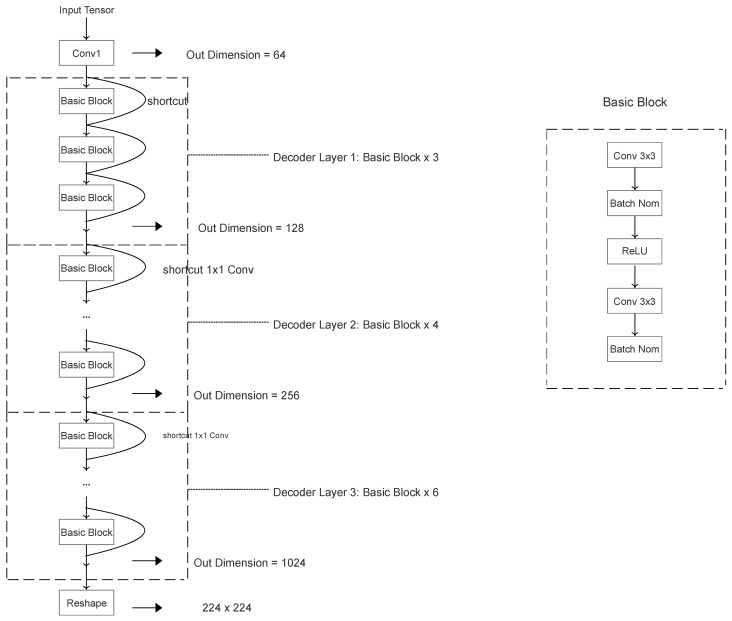
The proposed Decoder architecture. The liveness and spoofing Clue Maps generators are with the same architecture but with separate training parameters.

In Stage 2, the features are first reshaped to [12,56,56], which is for Decoders to generate corresponding Clue Maps. The Decoders are all ResNet-based structures, but with separate parameters to generate spoof or live Clue Maps. Specifically, the Decoders include one CBL module and 13 basic residual blocks, which are shown in Table 2. To illustrate the details of the Decoder structure, the designed ResNet-based decoder is shown in Figure 4 After obtaining the Clue Maps, we utilize Smooth L1 pixel-wise loss, because we assume that the live Clue Map should tend to be an all-zero map, and the spoof Clue Map tends to be an all-one map after model training. Additionally, considering strongly distinguishing the intra-class and inter-class samples, the second branch applies semi-hard triplet loss, which allows the distances between anchor and negative samples to be longer than the distances between anchor and positive samples. The bottom branch is a supervised learning branch only connected with Multi-layer Perceptron (MLP) for classification work. In addition, L2 regularization is applied to extracted features to avoid overfitting before the MLP. Thus, the final output of Stage 2 is to compute Smooth L1 pixel-wise loss, triplet loss and cross-entropy loss separately.

### 3.3. Loss Functions

For cross-entropy loss, as a traditional categorical loss function, its gradient calculation is much simpler, which leads to the faster convergence speed of the model in the training process and updates training parameters quicker in the limited training epochs. In contrast, using Mean Square Error (MSE) loss in logistic regression tasks could slow down convergence in classification due to its smoother gradient change, especially when it may produce a smaller gradient update in the early stage of training, and result in a slower training speed. Semi-hard triplet loss is a loss function commonly used in metric learning methods. It pushes the model to embed samples of the same class into the closer position of the feature space while separating samples of different classes from a theoretical perspective. In addition, this loss function largely prevents over-fitting, and its negative samples are slightly larger from the anchor than the positive ones. In this way, the model can avoid extremely difficult triples and excessive gradient changes. In conclusion, semi-hard triplet loss is effective in learning minor differences between samples by using “semi-hard” negative samples, and it could not only distinguish obviously different samples but also better capture the differences between samples that are visually similar but belong to different categories.

The embeddings obtained from the Transformer-based Encoder are represented by $f_{p}$ and $f_{n}$, where we regard them as live (positive) embeddings and spoof (negative) embeddings separately. Additionally, we want to ensure the anchor embeddings $f_{a}$ belong to the same class with positive embeddings and avoid manually grouping anchor, positive, and negative embeddings. We choose to generate positives and semi-hard negatives within a mini-batch, which is an online method. To distinguish intra-class and inter-class samples more effectively, we enforce that the Euclidean distance between the anchor and positive is shorter than the distance of the anchor with negative samples, but the positive exemplars with margin are further away from anchors than the negative samples. In other words, we let negative embeddings lie in the margin area to separate the minor differences between live and spoofs. The relationship between the embedding distances can be illustrated in Equation (2), and the triplet loss could be rewritten in Equation (3).

(2) d(a,p)<d(a,n)<d(a,p)+margin

(3) $$L_{tri}=\frac{1}{n}\sum_{i=0}^{n}\left[\right\|f_{a}-f_{n}{\|}_{2}^{2}-\|f_{a}-f_{p}{\|}_{2}^{2}-margin]$$

where *a* represents the anchor sample and $f_{a}$ is the anchor feature correspondingly; *p* represents the positive sample which belongs to the same class as the anchor and $f_{p}$ is the positive embedding; *n* represents the negative samples and $f_{n}$ is the negative embedding; margin is an enforced distance between positives and negatives.

In this work, we did not take an anomaly detection approach to design a one-class classification task for FAS or regard the live samples as a closed set. We would like to apply pixel-wise supervision of samples on the spoof set as well, so we assume that the “Clue Maps” should be an all-one or all-zero map ideally; for example, the generated Spoof Clue Maps $M_{S}$ should approximately tend to be an all-one map, and Live Clue Maps $M_{L}$ are all-zero maps after training. To measure the difference between generated “Clue Maps” and ideal “Clue Maps” (all-one or all-zero matrix) better, we choose the Smooth L1 loss to supervise the generated clues. This is because L1 loss is not smooth at the zero point, which means that the gradient derivative cannot be well performed. In addition, using L2 loss alone is more sensitive to outliers to cause the gradient explosion. Thus, we select Smooth L1 loss which combines the advantages of both losses, modifies the non-smoothing at zero-point, and is more robust to outliers. Specifically, the Smooth L1 loss for Spoof Clue Maps can be illustrated in Equation (4).

(4) $$L_{pixel}=\frac{1}{n}\sum_{i=0}^{n}\left\{\begin{array}{cc} 0.5{\left(M_{1}-M_{S}\right)}^{2} & if\;|M_{1}-M_{S}|<1\\ |M_{1}-M_{S}| & otherwise \end{array}\right.$$

where $M_{1}$ represents all-one map; $M_{S}$ represents generated Spoof Clue Maps.

Similarly, the Smooth L1 loss for Live Clue Maps can be illustrated in Equation (5).

(5) $$L_{pixel}=\frac{1}{n}\sum_{i=0}^{n}\left\{\begin{array}{cc} 0.5{\left(M_{L}-M_{0}\right)}^{2} & if\;|M_{L}-M_{0}|<1\\ |M_{L}-M_{0}| & otherwise \end{array}\right.$$

where $M_{0}$ represents all-zero map; $M_{L}$ represents generated liveness Clue Maps.

Finally, we design an auxiliary classifier to assist the model in making decisions, and cross-entropy loss is applied as a supervised learning method for model training. We only utilize the extracted features from Encoder with MLP to calculate the predicted matrix for spoofing and live images. Cross-entropy loss is used as one of the basic logistic regression factors for the final decision. The cross-entropy loss can be determined by Equation (6).

(6) $$L_{CE}=-\frac{1}{n}\sum_{i=0}^{n}\left[ylogp+\left(1-y\right)log\left(1-p\right)\right]$$

where *y* is the Ground Truth; *P* is the predicted probability of the class; *n* is the sample number.

In the final loss function design, we only want the CE loss to be used as an auxiliary loss function, while the triplet loss function and the pixel-wise loss function are used as the main loss measures to supervise the model to update the parameters. So, we combine these three loss functions linearly to obtain the final function in the end. Since the total loss is determined by three loss functions, we initially set the learnable weight balance for these three losses as 0.333. Then, the total loss function can be expressed as Equation (7).

(7) $$L_{tot}=\alpha L_{CE}+\beta L_{tri}+\gamma Lpixel$$

where α, β and γ are the initial learnable hyper-parameters.

## 4. Experiments

### 4.1. Datasets and Metrics

In this experiment, we used three datasets: CelebA-Spoof [23], OULU [22], and CASIA-MFSD [4]. CelebA-Spoof is one of the largest scale anti-spoofing datasets, containing 625,537 images of 10,117 subjects. The total number of live images is more than 202,599, which originated from the Celeb-A dataset; some subjects are filtered to guarantee the balance of spoof instruments. In addition, it is famous for its various diversity and four illumination conditions, and two environments are considered. Unlike other spoofing datasets, CelebA-Spoof provides 43 attributes with rich annotations, 40 of them are for live images and three attributes (spoof types, environments, and illumination) are well-labelled for spoofing images. Specifically, spoof types consist of paper print, paper cut, 3D mask, and replay attack, which are the most common attack methods in recent years.

The OULU dataset consists of 4950 real and attack videos, collected from different sensors, illuminations, and background scenes. Additionally, there are four protocols for evaluating the generalization ability, including unseen environment conditions of a PAD attack, the effects of different printers and displays, and the sensor’s interoperability. The dataset files define the category of data with the last string of the file names, including real faces (class 1), two paper attacks (classes 2 and 3), and two replay attacks (classes 4 and 5) separately.

Compared with CelebA-Spoofing, CASIA-MFSD is a small spoofing dataset that contains 600 video clips and 50 subjects for the training and testing phases. In each subject, there are 12 video clips, and only 1.avi, 2.avi, and HR1.avi belong to genuine classes shot by different cameras. In addition, we checked all the publicly available face anti-spoofing datasets, and found that they were all published on an older date. For example, the earliest dataset is Replay-attack published in 2012, and the CelebA-spoofing dataset was published in 2020. The main reason why we selected OULU-NPU, CASIA-MFSD, and Replay-attack is that most current works also utilize these datasets to prove their models’ generalization ability. Lastly, images and videos from these datasets are of good quality. Except for the Replay-attack dataset, other datasets contain various spoofing attack methods and different shooting cameras, which are adequate to cover and deal with face spoofing attacks in the real world. Thus, our research work also tests these datasets to compare with the previous benchmark results.

For evaluating metrics, we calculate the confusion matrix to obtain True Positive (TP), False Positive (FP), False Negative (FN), and True Negative (TN) separately first. Then, we utilize the Error Rate metrics including Attack Presentation Classification Error Rate (APCER), Bona fide Presentation Classification Error Rate (BPCER), and Average Classification Error Rate (ACER) to evaluate our approach performance and compare it with previous work. In addition, we also use Half Total Error Rate (HTER) as the cross-testing evaluating metric. The calculation is the same as ACER. The equations of APCER, BPCER, and ACER (HTER) are shown below:

(8) $$APCER=\frac{FP}{TN+FP}$$

(9) $$BPCER=\frac{FN}{TP+FN}$$

(10) $$ACER\left(HTER\right)=\frac{APCER+BPCER}{2}$$

where FP represents False Positives, TP represents True Positives, FN represents False Negatives, and TN represents True Negatives.

### 4.2. Implementation Details

For data augmentation, we choose the random data augmentation method, which contains four geometric data augmentations (Horizontal Flipping, Vertical Flipping, Random Resized Crop, and Random Rotation). These four methods [24] could generally perform better results than others in small-scale datasets. Since the fine-grained categories of spoofing (such as phone attack, paper attack, pad attack, etc.) have different sensitivities to colour, we do not apply colour transformations to prevent confusion among different fine-grained categories. For image processing work, all images are resampled and located in facial areas by SCRFD [25] with 0.5 NMS and enlarged by 1.2 of the bounding box. Then, we resize them to 3 × 224 × 224. For the training phase, we utilize the AdamW optimizer with a 0.0001 learning rate and 5 × 10^−4^ weight decay. There is a 0.2 dropout rate applied on the Linear layer to prevent overfitting. The triplet loss margin is set as 0.5, and learnable weight parameters of three loss functions are initialized as 0.333 in the first epoch. In addition, we select “swin tiny patch4 window7 224.pth” as pre-trained model parameters for Encoder, the batch size is 32 and the total training epoch is set to 30.

### 4.3. Ablation Test

Before we compared our model’s performance with other benchmark models, we performed a preliminary test to prove that the generation of “Clue Maps” with triplet loss and Smooth L1 loss is useful in live/spoofing classification work. There are three testing methods, which are the Swin Transformer with cross-entropy loss, the Swin Transformer with triplet loss and cross-entropy loss, and our proposed method. Specifically, the Swin Transformer architectures (as the Encoder part) are totally the same in this preliminary experiment. The triplet loss is applied to the extracted features of the Encoder part in the second testing method, but our method applied the triplet loss and Smooth L1 loss on the “Clue Maps” (Decoder part). In addition, we utilized CelebA-Spoofing as the intra-training set, and we randomly selected 20,000 images for training and 5000 images for validating. Then, we compute accuracy, APCER, BPCER, and ACER separately within 30 epochs, and the results are shown in Table 3. The updated hyper-parameters of loss functions’ weights are 0.27 for cross-entropy loss, 0.45 for semi-hard triplet loss and 0.28 for Smooth L1 loss, respectively. This preliminary experiment illustrates that the supervised learning method for anti-spoofing is not the most effective, adding triplet loss on Encoder will slightly increase the APCER, which means the model will wrongly predict more spoof samples, but the BPCER drop by 6% approximately, thus the combination of cross-entropy and triplet loss on Encoder could enhance the validating accuracy and classification ability, especially on the live sample set. Finally, our approach only utilizes cross-entropy loss as an assisted supervision loss function, which is applied on the auxiliary classifier, and the pixel-wise loss and triplet loss are all applied on the generated “Clue Maps”, then all Error Rates decrease largely, and the validating accuracy is significantly increasing as well. Thus, it is feasible to restore the features extracted from the Encoder to the “Clue Maps” of the original input size, and the unsupervised loss which applies to the Decoder part could both increase the classification ability on spoof and live sample sets.

### 4.4. Intra-Dataset Experiment

We carry out intra-dataset experiments on the first two protocols of OULU-NPU. For Protocol 1, the methods we compare the performances include the CNN baseline, CNN + MIL [26], GRADIENT [27], STASN [28], and FaceDS [29]. The results of APCER, BPCER, and ACER of OULU-NPU Protocol 1 are shown in Table 4. Our method outperforms all compared anti-spoofing methods on the first protocol to a large extent. Additionally, we also investigate one hand-crafted feature extraction method LBP’s performances; it could reach 5.0% APCER, 20.8% BPCER, and 12.9% ACER, respectively. Thus, we can conclude that the traditional CNN classification algorithm and manual feature extraction are gradually losing their competitiveness in this task.

For Protocol 2, it focuses on different spoofing attack methods. Thus, we select CNN baseline, GRADIENT, STASN, FaceDS, and Auxiliary [30] as the state-of-the-art models, and compare their relative Error Rate with ours. The validating results are reported in Table 5. Our model has slightly worse BPCER on this protocol, but could reach the lowest APCER and ACER, which are 1.5% and 1.6%. The STASN method has the lowest BPCER among the comparing methods.

### 4.5. Cross-Dataset Experiment

To demonstrate our model’s generalization ability, we set up several cross-dataset experiments. Firstly, we use the largest spoofing dataset CelebA-Spoofing as the training set and then test on Protocol 1 and 2 on OULU-NPU. The ACER results are 13.7% and 19.0%, respectively. We have found that our algorithm is slightly less effective at a wide range of forgery attacks (Protocol 2). However, the versatility of our model is relatively good, and the stability of recognition is high in the case of multiple scenarios (Protocol 1). Furthermore, we utilize CASIA-MFSD and Replay-attack [31] to perform cross-dataset experiments, because it is the current benchmark result which is widely used in the academic research field. Table 6 presents the cross-testing HTER of the previous methods. Our proposed method reduces the Error Rate by 3.6% in the first cross-testing experiment but increases by 9.4% on the second cross-testing HTER.

We reviewed the recently published papers, and found out they utilize different protocols to prove their models’ abilities to process unseen data. To ensure that our experimental data, protocols, and comparison methods are consistent, we used works that are relatively consistent and published in 2020. In addition, these selected works all used the same training and test datasets and protocols, which helped us to achieve a fair comparison. Furthermore, some of the recently published works did not publish their code. We aim to reproduce their code and compare our work with them as part of our future work.

## 5. Conclusions and Future Work

We reformulated the Face Anti-Spoofing task in an Auto-Encoder architecture with three supervised losses. The main innovation is to design a “Clue Maps” generator that is also the Decoder part of the whole network. Since there is less work to solve the FAS problem by using the Swin Transformer as a feature extractor, our work first shows that it could extract more useful information to a certain extent. Furthermore, Smooth L1 pixel-wise loss and triplet loss applied to the generated “Clue Maps” could help the model update parameters more efficiently and complete the liveness and spoofing classification task. This paper also identifies the importance of Decoder and the combination of Smooth L1 loss and semi-hard triplet loss in the ablation test. Meanwhile, we conduct extensive experiments on popular anti-spoofing datasets such as CelebA-Spoofing and prove our model’s generalization ability. Finally, we hope our generated Clue Map method could be useful for further investigators to complete more comprehensive contrastive learning and achieve better FAS performances.

Future works have two main aspects. The first aspect is to investigate whether fine-grained classification could outperform binary classification work. Due to the gradual diversification of the counterfeiting methods of spoofing and the categories of sensors, the fine-grained attribute classification task may help the model learn more spoofing information to a certain extent. With the differences in the shooting environment and shooting equipment of the samples, we also believe that the fine-grained attribute classification task of FAS will be a research trend in the future.

The second aspect is to develop an efficient and lightweight network for extracting the depth information for the training samples, which we hope to apply to the pre-processing work of the entire architecture. For the task of biological detection of living human faces, the extraction of depth map information can be achieved by computer vision technology. From previous experiments, it can be confirmed that the multi-modal detection task containing depth map information often greatly improves the testing metrics. A commonly used hybrid learning architecture is shown in Section 2.2, and the obtaining of depth maps with proper feature fusion methods is required to analyse in future works.

Face anti-spoofing is mainly used to unlock an individual’s financial system, but most liveness detection applications are still applying interactive FAS detection. For example, the testers follow the random instructions on the screen (such as closing eyes or shaking heads) to perform specific actions to determine whether the interacting individual is live or spoofing. Thus, our proposed silent FAS method can be used as an auxiliary tool in practical scenarios. Compared with other silent FAS methods, our method needs to be trained and tested in more scenarios and protocols. Since ViTs often require a large amount of data for training to ensure the excellent recognition ability of the model, we hope to fuse some datasets in future work and study the effect of cross-dataset data. In addition, our work does not include situations where multiple face images are used as input in data pre-processing. This issue may cause the recognition system to misjudge the face in the background, resulting in a degraded FAS performance.

## Figures and Tables

**Table 1:** The comparison of different EER methods. The Swin Transformer only utilizes cross-entropy loss to make binary classification.

Methods	EER (%)
Fine-tuned VGG-Face [14]	5.20
CNN [20]	6.20
Colour Texture [21]	6.20
Patch-based CNN [11]	4.44
Depth-based CNN [11]	2.85
Swin Transformer	4.77

**Table 2:** The Decoder’s components. Each Basic block contains two convolutional layers with kernel size 3, and strides are all set as 2.

Layer Name	Output Size	Layer Components
Decoder Conv1	64×28×28	2×2, stride = 2
Decoder Basic Block1	128×28×28	[3×3,3×3]×3
Decoder Basic Block2	256×14×14	[3×3,3×3]×4
Decoder Basic Block3	1024×7×7	[3×3,3×3]×6
Reshape	224×224	None

**Table 3:** Preliminary validating results for three methods. The training and validating data are from CelebA-Spoofing.

Methods	Epoch	Accuracy (%)	APCER (%)	BPCER (%)	ACER (%)
Method 1	18	86.35	4.444	17.672	11.507
Method 2	22	89.75	7.401	11.494	9.448
This work	17	93.52	2.184	8.773	5.479

Method 1: Swin Transformer with cross-entropy loss; Method 2: Swin Transformer with cross-entropy and triplet loss.

**Table 4:** The intra-dataset validating results for five compared methods. This table only presents the relative performances of the Protocol 1 scenario of OULU-NPU.

Methods	APCER (%)	BPCER (%)	ACER (%)
CNN (baseline)	7.8	22.3	10.1
CNN + MIL	3.3	9.2	6.3
GRADIENT	1.3	12.5	6.9
STASN	1.2	2.5	1.9
FaceDS	1.2	1.7	1.5
This work	0.9	1.5	1.2

**Table 5:** The intra-dataset validating results for five compared methods. This table only presents the relative performances of the Protocol 2 scenario of OULU-NPU.

Methods	APCER (%)	BPCER (%)	ACER (%)
CNN (baseline)	7.6	2.6	8.1
GRADIENT	3.1	1.9	2.5
STASN	4.2	0.3	2.2
FaceDS	4.2	4.4	4.3
Auxiliary	2.7	2.7	2.7
This work	1.5	1.7	1.6

**Table 6:** The inter-dataset testing results for six compared methods. The second column uses the CASIA-MFSD database as the training set, and the Replay-attack as the testing set. The third column is training on Replay-attack and testing on CASIA-MFSD.

Method	CASIA-MFSD	Replay-Attack	Replay-Attack	CASIA-MFSD
Motion	50.2%		47.9%	
CNN	48.5%		45.5%	
LBP	47.0%		39.6%	
Auxiliary	27.6%		28.4%	
FaceDS	28.5%		41.1%	
Spoof Cues [32]	27.4%		**23.7%**	
This work	**23.8%**		33.1%	

## Data Availability

Data are contained within the article.
